# Medical assistance in dying legislation: Hospice palliative care providers’ perspectives

**DOI:** 10.1177/09697330211012049

**Published:** 2021-09-19

**Authors:** Soodabeh Joolaee, Anita Ho, Kristie Serota, Matthieu Hubert, Daniel Z Buchman

**Affiliations:** Department of Evaluation and Research Services (DERS) Fraser Health, BC, Canada; Iran University of Medical Sciences, Iran; University of British Columbia, Canada; University of California, USA; Centre for Health Evaluation and Outcome Sciences (CHÉOS), Canada; University of Toronto, Canada; University of British Columbia, Canada; Centre for Addiction and Mental Health, Canada; Krembil Research Institute, Canada; University of Toronto, Canada

**Keywords:** Hospice, end of life care, Medical Assistance in Dying (MAID), Canada, qualitative research

## Abstract

**Background:**

After over 4 years since medical assistance in dying legalization in Canada, there is still much uncertainty about how this ruling has affected Canadian society.

**Objective:**

To describe the positive aspects of medical assistance in dying legalization from the perspectives of hospice palliative care providers engaging in medical assistance in dying.

**Design:**

In this qualitative descriptive study, we conducted an inductive thematic analysis of semi-structured interviews with hospice palliative care providers.

**Participants and setting:**

Multi-disciplinary hospice palliative care providers in acute, community, residential, and hospice care in Vancouver and Toronto, Canada, who have engaged in end-of-life care planning with patients who have inquired about and/or requested medical assistance in dying.

**Ethical considerations:**

The research proposal was approved by University of British Columbia Research Ethics Board in Vancouver and University Health Network in Toronto. Participants were informed regarding the research goals, signed a written consent, and were assigned pseudonyms.

**Results:**

The 48 participants included hospice palliative care physicians (n = 22), nurses (n = 15), social workers (n = 7), and allied health providers (n = 4). The average interview length was 50 min. Positive aspects of medical assistance in dying legalization were identified at (1) the individual level: (a) a new end-of-life option, (b) patients’ last chance to express control over their lives, (c) patient and family comfort and relief, and (d) a unique learning experience for hospice palliative care providers; (2) the team level: (a) supportive collegial relationships, (b) broadened discussions about end-of-life and palliative care, and (c) team debriefs provide opportunities for education and support; and (3) the institutional level: (a) improved processes to facilitate the implementation logistics.

**Conclusion:**

While being involved in the medical assistance in dying process is complex and challenging, this study sheds light on the positive aspects of medical assistance in dying legalization from the hospice palliative care provider’s perspectives. Medical Assistance in Dying legalization has resulted in improved end-of-life conversations between hospice palliative care providers, patients, and their families. Improved communication leads to a better understanding of patients’ end-of-life care plans and bridges some of the existing gaps between hospice palliative care and medical assistance in dying.

## Introduction

In June 2016, the Parliament of Canada passed federal legislation that allows eligible Canadian adults to request medical assistance in dying (MAID).^1^ Currently, the legislation allows a physician or nurse practitioner to directly administer a substance that causes the death of the person who has requested it or provide eligible patients with a prescription for a substance they can self-administer to cause their death. This legislation aims to respect the personal autonomy of eligible patients seeking access to MAID while at the same time protecting vulnerable people and the equality rights of all Canadians.^2^ On 17 March 2021, changes to Canada’s MAID legislation became law. The law no longer requires a person’s natural death to be reasonably foreseeable as an eligibility criterion for MAID. However, the Bill would continue to prohibit MAID for individuals whose sole underlying medical condition is a mental illness.^3^

Hospice palliative care providers (HPCPs) play a key role in supporting individuals with life-limiting and progressive illness, including the capacity to assess and attend to an individual’s existential and spiritual needs and explore an individual’s motivations for requesting MAID.

There is an ongoing debate concerning the relationship between hospice palliative care and MAID. One of the founders of palliative care in Canada explains that the vision for palliative care “was never to intentionally hasten death.” Sinding argues that MAID is not and should not be part of palliative care.^4^ However, a guidance document for hospice palliative care (HPC) clinicians and volunteers published by the Canadian Hospice Palliative Care Association posits that MAID and palliative care can co-exist.^5^

It is important to navigate all aspects of MAID implementation in Canada to ensure the development of best practices and the use of appropriate eligibility criteria and safeguards. In a larger study, our team investigated MAID from a broader perspective, which indicates that healthcare providers encounter multilevel MAID-related challenges.^6^ In one study, physicians reported strained relationships with colleagues who conscientiously object to MAID, inadequate financial compensation for their time, and experiencing increased workload that led them to sacrifice personal time.^7^ In a qualitative study on nurses’ experiences with MAID, participants perceived that they did not have adequate resources to access palliative care services that might help alleviate patients’ suffering. Nurses in this study also reported challenges receiving support from their teams and issues accessing appropriate policies, procedures, and systems that could guide their practice. Finally, they reported that an insufficient number of assessors and providers were available to support the number of patients seeking MAID.^8^ There may be a lack of protections in place to prevent health practitioners from experiencing ethical dilemmas.^9^ Despite these challenges, there are also reports of healthcare practitioners who consider providing MAID to be rewarding work.^10^

Over 4 years have passed since the Canadian Supreme Court ruled that patients experiencing grievous and intolerable suffering have the right to ask for help with ending their lives. Yet there is still much ambiguity about the effect that this ruling has had on Canadian society. Although several studies have investigated healthcare providers’ opinions about MAID and challenges that they encounter, few studies have directly examined how frontline multi-disciplinary HPCPs describe the positive aspects of MAID legalization.

This article is part of a larger project that aims to understand the processes of navigating MAID from the standpoint of HPCPs. We describe the positive aspects of MAID legalization from the perspective of HPCPs who have engaged in end-of-life (EOL) care planning with patients who have inquired about and/or requested MAID.

## Methods

### Study design and procedures

In this qualitative descriptive research, we conducted semi-structured in-depth interviews in person or by phone with HPCPs in Vancouver and Toronto, between 2018 and 2020. The interviews conducted by or under supervision of a team member with extensive expertise is qualitative research. Most of the in-person interviews in Vancouver were conducted in a private room in the units where participants worked, while two participants invited the interviewer to their home for the interview. In Toronto, 10 interviews occurred in a private room in the clinic where the participant worked and 4 took place in a research office at Toronto. Eight interviews in Toronto were conducted over the phone at the participants’ request. We used purposeful sampling^11^ to recruit a range of multi-disciplinary HPCPs, including nurses, physicians, social workers, and other allied healthcare providers across acute, community, residential, and hospice care. All participants had experience engaging in conversations about EOL planning with patients who had made general inquiries or formal requests for MAID. We circulated recruitment materials through professional listservs, clinical presentations by the research team, and professional contacts. Interested prospective participants were contacted by a team member who confirmed eligibility and provided further information. A member of the research team sent consent materials to participants prior to their interview.

Data gathering started in Vancouver and then continued in Toronto. We developed the interview guide based on a literature review and by our research team’s practice experience. We piloted the interview guide with three HPCPs with experience responding to MAID requests and refined the interview guide according to their feedback. Interviews were audio-recorded, transcribed, and de-identified before analysis. Field notes documented the recruitment and interview contexts as well as participants’ speech and non-verbal behavior.

## Data analysis

Our multi-disciplinary research team conducted inductive thematic analysis with the initial data and engaged in constant comparison of indicators, concepts, and categories throughout the research process. Patton describes inductive thematic analysis as a method for identifying, analyzing, and reporting patterns (themes) within data; the identified themes are inductive because they are strongly linked to the data themselves.^12^ After reviewing each transcript and familiarizing ourselves with the data, we collaboratively developed a coding framework and discussed the application of these codes to the interview transcripts. Three research team members were involved in the coding process and analysis at each site. We utilized a constant comparative approach to systematically organize, compare, and understand the similarities and differences among participants’ perspectives.^11,13^ The concurrent and iterative process of collecting and analyzing the data facilitated the comparison between newly emerging themes and categories with those previously established in the dataset.^14,15^ This iterative process allowed us to determine when data saturation had been reached.^16^ Saturation occurred when no new themes emerged from further interviews.^11^ To promote analytic rigor and trustworthiness, researchers recorded analytic memos that documented self-reflections and provided a space to engage in a critical analysis of the emerging ideas.^11,16^ We used the NVivo 12 software to facilitate data management and analysis. We adhered to the Consolidated Criteria for Reporting Qualitative Research checklist.^17^ The research team included experts with strong backgrounds in bioethics, nursing, and qualitative methods.

## Ethical considerations

The research proposal was approved by University of British Columbia Research Ethics Board in Vancouver and University Health Network in Toronto. Participants were informed of the research goals, signed written informed consent forms, and were assigned pseudonyms. All identifiable information was removed from the transcripts to protect confidentiality.

## Results

The 48 multi-disciplinary participants from Vancouver (n = 26) and Toronto (n = 22) respectively included HPC physicians (n = 22), nurses (n = 15), social workers (n = 7), and other allied health providers (n = 4). Most participants identified as female, had over 10 years of work experience, and were employed in secular institutions (Table 1). The average interview length was 50 min (range, 30–97 min).

**Table 1.** Participant demographics.

Characteristic	Qualitative study sample, n = 48
Gender, n (%)	
Male	9 (19)
Female	39 (81)
Age range, n (%)	
25–34	8 (17)
35–44	15 (31)
45–54	17 (35)
55–64	6 (13)
65–74	2 (4)
Role, n (%)	
Physician	22 (46)
Nurse	15 (31)
Social worker	7 (15)
Allied health professional	4 (8)
Type of institution, n (%)	
Faith-based	13 (29)
Secular	32 (71)
Location, n (%)	
Community	1 (2)
Hospice	7 (15)
Hospital palliative care	26 (54)
Multiple sites	14 (29)
Work experience in years, n (%)	
<1	2 (4)
1–5	12 (25)
5-10	14 (29)
>10	20 (42)
MAID assessment and provision, n (%)	
Assessor	8 (16.6)
Provider	5 (10.4)
Neither	40 (83)

MAID: +medical assistance in dying.

Positive aspects related to MAID legalization were identified at (1) the individual level, (2) the team level, and (3) the institutional level.

### Positive aspects of MAID at the individual level

Hospice palliative care providers reported the most positive effects of MAID legalization at the individual level. Positive aspects of legalization reported at this level include positive interactions with individual patients, families, and healthcare provider colleagues. These positive individual effects have been divided into four sub-themes: the legislation allows patients to have a new EOL option that was not previously available, MAID allows patients to express control over their lives, MAID conversations can provide comfort and relief to patients and their families, and MAID legislation represents a new learning opportunity for HPCPs.

#### A new EOL option for patients

HPC specialists are trained to provide ongoing care until death, but sometimes despite doing everything, they are not able to alleviate patient’s intolerable suffering. Having MAID as an additional clinical care option available in the context of EOL care was described as positive:

you had palliative care, which is very effective and was still a great option, but you felt, like, you couldn’t necessarily address all of their concerns. And I guess post legalization, I would explore it the same way, and then now there’s actually an option for them to seek that out. (Pauline, MD, Vancouver)

This new EOL option was described as being especially important for younger adult patients who experience suffering from physical and/or emotional effects of the disease over their body. MAID allows them to have another clinical care option to explore in EOL care planning discussions:

…there are always situations, often with very young patients, their hearts and lungs and kidneys are very strong and resilient and it takes a long time for them to die.…. And what would have taken weeks more for a patient to slowly wither away in bed with very poor quality of life now…I think that’s better because we still have all the options we had before, plus we have an option that…it feels more honest to me than just putting someone on a drip to make them…insensible…. (Kim, MD, Toronto)

#### Patients’ last chance to express control over their lives

One of the most salient positive aspects described by HPCPs at the individual level was the ability to use MAID as a means of respecting patients’ autonomy. Legalization allows patients who choose this option to have increased control over their EOL journeys:

*I* feel like the MAID provision is a person’s last act of empowerment, that while the disease or whatever they’re suffering from has taken so much from them, you know, that this is their last hurrah, their last wish, and, yeah, that they were heard and listened to and able to go in the way that they chose. (Megan, SW, Vancouver)

Losing control over everything else makes MAID the last chance patients have to control how they may want to end their life. One participant described,

…for the people who have lost so much over everything else, this might feel like something that they do have some control and they find that to be positive. (Andrea, MD, Toronto)

#### Patient and family comfort and relief

In addition to individual control, participants emphasized the comfort and relief that MAID brings to the patients who request it:

…it just provides the patient with so much relief. It’s sometimes quite remarkable…even if they just want to know more information about it, just the confirmation that it is available to them seems to provide a lot of relief and seems to sort of decompress them in some way and so then they actually are more comfortable…. (Valerie, MD, Toronto)

Some HPCPs had difficulty articulating the complex emotions they experience when supporting patients through the MAID process, from the initial conversation, through the formal request, to provision:

…it’s been really nice because like the family has really been able to be like “Okay, this is when it’s going to happen so we’re all going to get together like a few hours before. We’re going to hang out. We’re going to spend time together. And they feel like there’s a lot of closure around it. And the patient felt very relieved and I would also say content. Not sure happy is the right word, but content and looking forward to her perceived suffering, ending. (Robin, SW, Toronto)

I think for me it is the resolve that people have. So, I think just that, the majority of the people seem so ready and so at peace. It’s quite something to see that. Knowing their suffering is over, and that relief that people have, it’s quite something to witness. I think…I think that’s a big part of it. (Sophia, SW, Vancouver)

Based on participants’ experiences, this feeling of comfort was even seen in some families’ reaction in response to the patient’s decision and feeling of appreciation that their loved one is no longer suffering.

#### A unique learning experience for HPCPs

HPCPs described how their involvement in the MAID process represented a unique learning experience. Participants explained how they have learned that each MAID provision provides ample learning opportunities:

…like I said, no two are the same. Every family reacts differently, every patient reacts differently, especially on our unit here because we’ve had so many, it’s really…I don’t want to say it’s become the norm, but we’ve become so practiced at it, it’s just like it’s a part of the day, and yeah, we reflect afterwards and we make sure everybody’s okay. (Nina, RN, Vancouver)

When the first person who requested and received assisted dying in our organization went through with it, it was a complete paradigm shift…And we call her our greatest teacher because she was so clear in what she wanted and actually guided us as a healthcare team. Patients themselves and most family members are incredibly generous to let me bear witness to their process because it’s huge learning for me and it’s just a privilege to be at the bedside when someone’s going through this, both as a clinician and a researcher…. So I’m learning from every patient that I’ve worked with, with regard to MAID. (Gwen, Allied healthcare professional, Toronto)

### Positive aspects of MAID legalization at the team level

Developing safe and sustainable working conditions for HPCPs involved in the MAID processes is important for enhancing MAID accessibility for patients. The positive aspects of MAID legalization that HPCPs described at the team level include examples of (a) supportive collegial relationships, (b) broadened discussions about EOL care planning within palliative care, and (c) team debriefs provide opportunities for education and support.

#### Supportive collegial relationships

Participants described the positive impacts of engaging in supportive relationships with their colleagues. HPCPs described how they enjoyed sharing in mutually supportive relationships with their colleagues and working with multi-disciplinary teams to assist patients seeking MAID. These positive team dynamics made HPCPs feel supported and encouraged them to continue engaging in MAID-related work:

I think also what’s been very rewarding is being able to work with great colleagues through this (MAID) and just being able to continue to be supportive of everybody, even though they may have very diametrically opposite opinions about how someone’s end-of- life should happen. (Stella, RN, Toronto)

These positive collegial relationships and supportive multi-disciplinary teams include physicians, nurses, social workers, spiritual care providers, ethicists, and allied health professionals across many healthcare areas. Effective teamwork and mutually supportive inter-disciplinary relationships lead to better communication among healthcare providers and improved patient care. These benefits were described by HPCPs who are part of established MAID teams and those who identified as being new to this area of practice. These benefits were also identified by those who do not participate directly in MAID assessments or provisions, including HPCPs who describe themselves as patient advocates, and those who refer patients seeking MAID to the appropriate teams and resources:

…I’m fortunate in the group I work in that was worked out before I came onboard. So they’d worked out their process and they’d been doing and…so I’m the first to acknowledge I’m the new kid to that group, but it seems to work rather well. Within our team, we have a group of people who provide MAID. We have a group of people who do not, but do assessments, and we have a group of people who don’t do either but they refer into the group who do. (William, MD, Toronto)

#### Broadened discussions about EOL and palliative care

Despite different values and opinions that HPCPs have toward MAID, many participants described the positive space that MAID discussions created in EOL discussions. The ability to speak openly about MAID benefits not only patients interested in discussing this EOL option but also opens space to have constructive conversations about broader EOL care planning within diverse HPC teams:

I think, how thoughtful people are in understanding what their patients are wanting and needing. And I think also our team has become increasingly open to taking on these cases and just trying to understand what it is that they need and could benefit from. (Whitney, MD, Toronto)

They believed those conversations also create a better space to talk about other palliative care options:

…People should be supported, in their choices, and that they should be given full information as to what their options are. Well, before it wasn’t really talked about.…Now, when people request MAID, it does need to be confirmed that they have had counseling; in terms of what their palliative care options are. So, I think that’s really important as well that people don’t run towards it just because they want to take control, and avoid their pain, but that they really know yet what palliative care options are as well. If they don’t choose this, so. (Ellie, SW, Vancouver)

Participants believed these supportive conversations provide space for patients to consider all EOL care options available to them so that they can make well-informed decisions about whether they want to pursue palliative care, MAID, or both.

#### Team debriefs provide opportunities for education and support

HPCPs who engaged in team debriefings for MAID cases were satisfied with these meetings’ learning and support opportunities:

We have nursing support ready; we have a debriefing before and after the MAID, just for staff as well as for the family because we have a lot of conscientious objectors that work here as well, so we want to make sure everybody is supported. (Nina, RN, Vancouver)

The positive aspects of team debrief include having an opportunity to share unpleasant or challenging feelings that come up surrounding the MAID case and being able to receive support from colleagues:

I think one of the things that I am so thankful for is a training that really has left me deeply convinced of the benefits of a debriefing. And so, whenever I have a patient that leaves me upset, maybe perhaps they were yelling at me or you know, that likewise, I would debrief about that case just like I would debrief about a MAID case. (Judy, SW, Toronto)

These meetings also provided opportunities for participants to learn more about the MAID processes and procedures at their institutions.

### Positive aspects of MAID legalization at the institutional level

From HPCPs’ perspectives, the majority of MAID’s positive institutional-level effects were related to the development and continued improvement of processes that support patients’ EOL choices.

#### Improved processes facilitate implementation logistics

HPCPs described how the institutional logistics related to MAID conversations, referrals, assessments, and provision have improved in the years since legalization. Improved logistics at the institutional level facilitate MAID processes and make this option more accessible to patients seeking this EOL option. However, despite these positive changes, challenges related to MAID logistics and resources persist:

…all across the region, all the teams, have kind of developed and have gotten much better at pre-screening, bereavement risk assessment tools, having a much clearer idea sort of what might go wrong. The nurses have gotten better at doing the IVs, physicians have gotten better at doing their assessment so that there aren’t issues about competency. Everybody’s kind of just, you know, with time and experience and knowledge, we’ve all kind of upped our game. (Anna, SW, Vancouver)

Participants described the extensive work they have done to prepare themselves and their institutions to respond to MAID requests. Now, many feel well situated to accept these requests:

…we had undertaken a fairly extensive process as an organization, as a group, in anticipation that MAID was going to become legalized. So, we, sort of being on the frontlines of this, had a sense that when the court agreed to hear the case, we started to think, “We better figure out what we’re going to do, ’cause things could change very quickly,” right?…So, when the court ruled, we actually weren’t…didn’t come as a shock or a surprise or, like, “Oh my God, what are we going to do now?” (Henry, MD, Toronto)

Frontline HPCPs took up leadership roles within their institutions by serving on committees that developed, applied, and evaluated MAID policies and processes:

I sat on that committee, helped with the policy, helped in terms of figuring out if our division, how would we fit into that, how would we safeguard the palliative work as separate and distinct from MAID but still partner with it in a way that we would ensure that our patients and patients who we hadn’t met but should be meeting were getting connected…. (Olivia, MD, Toronto)

## Discussion

Many researchers have investigated the complex challenges that have resulted from the government of Canada’s decision to legalize MAID.^8,9,18–20^ Our broader study also discovered that participants encountered many challenges, ethical dilemmas, and moral distress in navigating MAID.^6^ Shaw et al.^10^ reported physicians’ rewarding experiences providing MAID in British Columbia. Nonetheless, to our knowledge, this is the first article to report the positive aspects of providing MAID-related services from the perspectives of multi-disciplinary HPCPs. Our findings discussed the positive aspects of MAID legalization at three interconnected levels: within personal experiences, multi-disciplinary healthcare teams, and institutions.

At the individual level, HPCPs described how a patient’s choice to have MAID represents the last chance for them to express control over their lives. Participants perceived this as being positive not only for the patient and their family members but also for themselves as they were able to actively support the patient in fulfilling their EOL wishes. For many participants, being able to facilitate a patient’s achievement of their desired EOL plans meant providing high-quality patient-centered care which brought them satisfaction.

Some studies that have directly examined patients’ perspectives regarding MAID also reported autonomy and control as key factors motivating patients’ EOL decisions.^21,22^ In a report by the Oregon Public Health Division, people who requested MAID cited loss of autonomy as one of their primary EOL concerns.^21^ In addition, in a study exploring the experiences of people who pursued MAID in Vancouver, the importance of having autonomy and control over one’s EOL was the most prominent theme. Participants in this study wanted to decide for themselves when their suffering was too great and they wanted access to EOL options that included MAID.^22^ For some of these patients, choosing MAID provided them with a sense of relief, as they see this as a way of ending their unbearable suffering. Some patients described planning gatherings before their MAID intervention so that their last moments can be spent surrounded by family and friends.

Nurses have also reported supporting and enabling patients to have a good death through MAID. For these nurses, participating in MAID has reinforced their role as comfort care providers who are able to mitigate patients’ suffering. Participating in MAID has broadened their understanding of what a so-called “good death” could be.^19^ In a qualitative study interviewing nurses, social workers, and pharmacists with firsthand experience delivering MAID at an academic hospital in Toronto, participants considered MAID as a form of care for patients, families, other healthcare workers, and even themselves. As one of the nurse participants said, “even though it’s so tough, at the end of the day, I go to bed knowing that person’s suffering is done, and I think that gives me peace. Especially because it’s what they want as well.”^23^ Nurses in Beuthin et al.’s^19^ study found it difficult for them to effectively articulate the paradoxical experience of witnessing MAID deaths as they are simultaneously “sad” and “beautiful.” In our study, similarly participants reported experiencing complex emotions involved in caring for patients requesting MAID.^6^ Despite these challenges, most participants told us that they find comfort in witnessing patients’ relief from suffering.

In a case study by Quinn and Detsky, the authors describe their experience providing MAID to a 64-year-old patient with gastric cancer. In a report of the lessons they learned from the case, they said,

we learned when many of our other patients died, the hardest part for the family was dealing with the uncertainties; when will they die, how would they die, what will it look like, is he or she in pain? And they have to make serial decisions; when to stop blood work, intravenous hydration, vital signs, and remove tubes? All of these uncertainties and decisions induce enormous distress. With MAID, all of that uncertainty and agonized decision making is removed. As a result, the family and the patient undergo much less stress.^24^

Now that Canada has legalized MAID, patients can pursue a range of options to alleviate their suffering at the EOL. These options include both palliative care and an assisted death.

In our study, multi-disciplinary HPCPs described the positive aspects of allowing patients to access MAID, which some of them considered to be “another clinical care option at the EOL.” Participants thought that it was important for patients to be able to exercise choice at the EOL and actively engage in EOL care planning. They reported that despite all of their efforts to deliver effective palliative care, it is not always possible to address all of a patient’s concerns and relieve his or her suffering. A desire for MAID appears to be particularly salient for younger adult patients who are suffering from long-term physical and/or emotional effects of disease.

Participants considered MAID to present a unique learning experience that was unlike the education they had received previously. Throughout the entire process, learning took place through having conversations with patients and family members, attending and participating in MAID provision, and working on multi-disciplinary teams. This learning enabled HPCPs to better support patients and families, their teams, and themselves.

At the team level, mutually supportive collegial relationships and effective teamwork were described as positive and rewarding. For example, one of our participants explained how “strong teamwork” was vital to promote the MAID process. Participants described how they built relationships with encouraging and supportive colleagues. While MAID requires teamwork, there are provincial differences in regard to how MAID is enacted.^1^

Participants perceived that the legalization of MAID created more opportunities for broader EOL conversations with patients receiving HPC and among healthcare teams. Improving conversations about EOL options, including MAID, creates opportunities for HPCPs to discuss various EOL options with patients and ensure that resources and information are accessible so that they can make well-informed decisions about their care. Engaging in continuous conversations about EOL planning, including MAID, is important as new perspectives and considerations can arise through dialogue between patients, their loved ones, and their healthcare providers.^25^

Our participants explained that working with strong, supportive multi-disciplinary teams is necessary for the successful implementation of the MAID legislation. Many participants in our study reported that multi-disciplinary MAID debriefs are a vital source of education and support for HPCPs. The results of a 2017 Canada-wide survey of Canadian Hospice palliative Care Association (CPHCA) members including nurses, administrators, physicians, and volunteers indicated participant dissatisfaction with the current psychological and professional support being provided by healthcare institutions and the Ministry of Health during and after MAID procedures.^26^ The inclusion of these debriefs in various institutions may be the results of MAID process improvement initiatives that have occurred in many EOL care contexts since legalization.

Debriefing was described as a useful support tool for the multi-disciplinary participants in this study. The *Medical Assistance in Dying (MAID) Protocols and Procedures Handbook* recommends holding debrief sessions for healthcare providers who are involved in MAID processes. Debriefs are listed as a procedural recommendation for all healthcare providers, but especially for those who are new to MAID. During the debrief, care providers should reflect on their emotions. This can be done with other MAID providers, trusted colleagues, or a spouse or partner.^27^ These debrief protocols emphasize the importance of supporting the MAID team. A scoping review on the relational influences of assisted dying experiences reported that the need for debriefing has been described in many studies.^28^ Debriefs may strengthen relationships among physicians who practice MAID as these conversations provide opportunities for colleagues to confide in and learn from each other.^29^ Previous research also indicates that nurses describe debriefing as a coping strategy that helps them deal with MAID.^30,31^ Our study suggests that debriefing also has a vital role in supporting multi-disciplinary health professionals engaging in MAID conversations, referrals, assessment, and provision. Given these findings, debriefing should be incorporated into the MAID process in a variety of healthcare settings.

At the institutional level, participants suggested that MAID processes have improved over time. The provision of MAID within healthcare facilities imposes specific institutional obligations to ensure effective and appropriate delivery of the intervention. Institutional policies support and protect the rights of patients, families, and healthcare providers and provide education for patients seeking information about MAID. Institution-based delivery and the hospital-wide education processes surrounding it have brought assisted dying more prominently into the public space of medical care. These circumstances have enhanced transparency and accountability regarding the range of medical practices at the EOL and have encouraged more open conversations about wishes, fears, and preferences.^32^ In our findings, that participants mentioned open conversations about EOL options at the team and institutional levels creates a more effective and supportive space to talk about all HPC options, including MAID. It has been noted that such open conversations, which has been called “public dying,” may help to diminish stigma and fear about the EOL. Public dying may also reduce barriers of silence and isolation that may exist between dying people and the worlds in which they belong.^32^ In a qualitative study exploring the perspectives of family caregivers of patients who underwent MAID in Toronto, Hales et al.^33^ reported a lack of clarity regarding who would be involved in the MAID process, including whether there was a “MAID team” to coordinate the process. Some Canadian institutions anticipated MAID’s legalization and immediately began working to determine how to translate the legal ruling into practice. Our results suggest that this early planning had positive effects on PHCPs working in these institutions. Conversely, participants who worked in institutions that did not have clear MAID policies and procedures, dedicated MAID teams, and MAID coordinators experienced more significant challenges than those who did have these teams built into their hospitals. As time passed during our research data collection stage, MAID processes and systems became more established. Our yyy participants, who were interviewed several months after the xxx cohort, reported greater clarity and smoothness than xxx in their institutions’ MAID policies and procedures. In Ho et al.’s^6^ report on the challenges experienced by HPCPs in Vancouver, participants discussing multilevel MAID-related challenges reported gradual improvements in organizational policies and coordination over time. We acknowledge that the challenges that HPCPs encounter are nuanced and complex, and a few of our participants, especially those who reported being against the MAID legislation, did not report positive aspects except potential engagement of EOL conversations. However, it is also integral to examine how HPCPs conceptualize the positive effects of MAID’s legalization at the individual, team, and institutional levels.

## Strengths and limitations

Having participants from diverse multi-disciplinary contexts provides a maximum variation in training and experience that makes this study more rigorous. Despite this occupational diversity, most HPCPs in our study identified as female (reflecting the gender makeup of HPCPs) and were generally supportive of the MAID legislation. Thus, the transferability of our findings may be limited to HPCPs of similar demographics. In addition, the results of this study reflect HPCP’s experiences, perceptions, and recollections. To establish a more fulsome understanding of the positive impacts of MAID, further research should be examining patients and families’ experiences. Analyzing MAID’s impact from multiple perspectives may allow for a more holistic understanding of how MAID conversations, assessments, and provision are experienced differently for patients and family members.

## Conclusion

This is the first article to focus specifically on the positive aspects of MAID legalization from the perspectives of multi-disciplinary HPCPs in Canada. Our study illuminates the positive aspects of MAID on three levels: the individual level, the team level, and the institutional level. Participants acknowledge that while being involved in MAID conversations referrals, assessments, and provisions can be challenging and complex, there are also positive aspects of engaging in these processes that provide satisfaction. These positive experiences include having more open conversations with patients and family members about EOL care planning, exploring various EOL options including MAID, and having the ability to ameliorate the suffering of patients who choose MAID as an EOL option.

## Supplemental material

Supplemental Material, sj-doc-1-nej-10.1177_09697330211012049 - Medical assistance in dying legislation: Hospice palliative care providers’ perspectives Click here for additional data file. Supplemental Material, sj-doc-1-nej-10.1177_09697330211012049 for Medical assistance in dying legislation: Hospice palliative care providers’ perspectives by Soodabeh Joolaee, Anita Ho, Kristie Serota, Matthieu Hubert and Daniel Z Buchman in Nursing Ethics
