# Supplementary material for: Medical assistance in dying legislation: Hospice palliative care providers’ perspectives
Source: Nurs Ethics. 2021 Sep 19;29(1):231–44. doi: 10.1177/09697330211012049 (PMC8866752; doi:10.1177/09697330211012049)
Supplement: Supplemental Material, sj-doc-1-nej-10.1177_09697330211012049 - Medical assistance in dying legislation: Hospice palliative care providers’ perspectives [file sj-doc-1-nej-10.1177_09697330211012049.doc]

**Interview Questions**

1. We would like to hear a bit about your general experience engaging in EOL care discussions and providing EOL care before and after MAiD became legal.

1. When you first heard about MAiD possibly becoming a legal option, what came to your mind regarding your role as a PHCP? What are your opinions of patients who have requested MAiD being cared for on hospice and palliative care wards or facilities?

1. How would you compare your roles and responsibilities before and after MAiD became legal? (e.g., Has MAiD had any impact on who initiates EOL conversations, how you or your colleagues engage in EOL discussions?)
2. Some people believe MAiD is another clinical tool or care management plan for patients at the EOL, whereas others think that they are in different categories. What are your thoughts about MAiD in that regard? (Follow up prompt: How does your current view on that compared to the pre-MAiD era?)
3. Can you tell me about the first time you encountered or heard about a patient inquiring about MAiD at your workplace, or an experience caring for a patient who had requested MAiD that has stuck with you?

Prompts:

- 1. What was the patient’s health situation? Socio-economic status? Family/Relational contexts? Where was the patient located?
  2. Who initiated the conversation, and how did the issue come up?
  3. Known or suspected reasons for the patient’s inquiry?
  4. Family’s responses/engagement in the process?
  5. How did the patient die in the end?
     1. If they passed away before date of provision, from your perspective, how was the EOL experience for the Patient? Family? Provider?
  6. Your personal thoughts about the patient’s decisional process and/or the decision itself?
  7. How was the process of referring, assessing, or providing MAiD? (e.g., personal, institutional, and societal resources available/utilized)
  8. In your most recent case, how was it similar to or different from other MAiD experiences?

1. Can you tell me a bit about your experience responding to general inquiries or formal requests for MAiD?

Prompts:

- 1. What is your approach to this conversation (e.g. questions you ask/ topics you explore, resources you use or refer to)?
  2. What were some surprising things about those situations?
  3. What were the most positive aspects from those experiences? (e.g., personal strengths, institutional/systemic resources)
  4. What were the most challenging aspects of those experiences (for you, the team involved, or the patient/their family)? (Prompt for morally distressing or ethically challenging issues.)
  5. How may your personal background or characteristics (e.g., cultural and educational backgrounds, professional role, personal history) influence how you manage MAiD requests and/or care for these patients? In what ways?
  6. Has your professional education/training provided you with the skills and resources to deal with these situations? In what ways?
  7. Have you encountered situations that have raised interprofessional team conflict/disagreement related to a MAiD request/assessment/provision?

1. [If the participant is an assessor or provider:
   1. Can you tell me about your decision to become an assessor/provider?
   2. If an assessor, have you considered becoming a provider?
   3. (Refer to section P), Do you have any recommendations to improve/enhance providers’ training and education in navigating MAiD process?]
2. The objectives of our project include designing resources and programs to support PHCPs in delivering EOL care in the post-MAiD era and determining future directions of research.
3. What are some additional resources or training that would help support you to care for patients who inquire about MAiD and their families?
4. What are some areas of research regarding MAiD and palliative/hospice care in the post-MAiD era that you think should be explored further?
